# Simultaneous EEG Acquisition System for Multiple Users: Development and Related Issues

**DOI:** 10.3390/s19204592

**Published:** 2019-10-22

**Authors:** Sunghan Lee, Hohyun Cho, Kiseong Kim, Sung Chan Jun

**Affiliations:** 1 School of Electrical Engineering and Computer Science, Gwangju Institute of Science and Technology, Gwangju 61005, Korea; sunghanlee@gist.ac.kr; 2 Wadsworth Center, New York State Department of Health, Albany, NY 12201, USA; Hohyun.Cho@health.ny.gov; 3 Department of Bio and Brain Engineering, Korea Advanced Institute of Science and Technology, Daejeon 34141, Korea; 4 R&D Center, BioBrain Inc., Daejeon, 35203, Korea

**Keywords:** EEG, hyperscanning, simultaneous acquisition

## Abstract

Social interaction is one of humans’ most important activities and many efforts have been made to understand the phenomenon. Recently, some investigators have attempted to apply advanced brain signal acquisition systems that allow dynamic brain activities to be measured simultaneously during social interactions. Most studies to date have investigated dyadic interactions, although multilateral interactions are more common in reality. However, it is believed that most studies have focused on such interactions because of methodological limitations, in that it is very difficult to design a well-controlled experiment for multiple users at a reasonable cost. Accordingly, there are few simultaneous acquisition systems for multiple users. In this study, we propose a design framework for an acquisition system that measures EEG data simultaneously in an environment with 10 or more people. Our proposed framework allowed us to acquire EEG data at up to 1 kHz frequency from up to 20 people simultaneously. Details of our acquisition system are described from hardware and software perspectives. In addition, various related issues that arose in the system’s development—such as synchronization techniques, system loads, electrodes, and applications—are discussed. In addition, simultaneous visual ERP experiments were conducted with a group of nine people to validate the EEG acquisition framework proposed. We found that our framework worked reasonably well with respect to less than 4 ms delay and average loss rates of 1%. It is expected that this system can be used in various hyperscanning studies, such as those on crowd psychology, large-scale human interactions, and collaborative brain–computer interface, among others.

## 1. Introduction

Social interaction is one of the hallmarks of people’s daily lives, as humans have lived in societies and interacted with each other since prehistoric times. Recently, some neuroscientists have studied social interactions from the neural activity perspective, and interest in interpersonal neural synchronization (INS) during social interaction has increased rapidly; research on neural mechanisms, interpersonal coordination, and certain related issues during social interaction is blossoming and is referred to as hyperscanning [1]. Dyadic interactions have been studied during various tasks, such as visual gaze-based interactions [2,3], verbal interactions [4,5,6], hand movements [7,8,9], the social game [10], and music performance [11,12,13], among others. In addition, they have been applied to estimate mutual cooperation levels to ensure proper efficiency and safety standards in operational environments [14,15,16]. Our neuroscientific understanding of social interaction has made great advancements in recent decades, and Phi complex [7] and alpha interbrain synchrony [6,17,18,19] have been reported as neural behaviors and markers in social interactions.

Human social activities in daily life include not only dyadic interactions, but also occur in the forms of many-to-many interactions (e.g., choruses, band performances, brainstorming, and discussions) and one-to-many interactions (seminars, lectures, etc.). However, most hyperscanning studies have been conducted only on dyadic interactions, and very few have investigated three or more people’s interactions. Several reviews of hyperscanning [16,20,21,22,23,24,25,26] have reported that most studies’ experimental designs were quite limited and there are few compelling group interaction studies. They emphasized near-realistic group interaction studies as one of the future directions for hyperscanning.

The brain imaging techniques used widely in hyperscanning studies include functional magnetic resonance imaging (fMRI) [27,28,29], functional near-infrared spectroscopy (fNIRS) [30,31], magnetoencephalography (MEG) [32], and electroencephalography (EEG) [33]. Given the scalability necessary to allow simultaneous multiple connections, fNIRS and EEG seem to have the best potential in that they are portable and can acquire data online from three or more people at a reasonable cost. Indeed, the recent hyperscanning literature shows that fNIRS and EEG have been used most often to acquire data from three or more interactors. In the case of fNIRS, Jiang et al. [5] reported the difference in INS between leader–follower pairs and follower–follower pairs during three-person group discussions, while Dai et al. [34] studied INS via the cocktail party effect [35] during three people’s group discussions. Furthermore, Nozawa et al. [36] conducted a cooperative word chain game (Shiritori) with a group of four people using two-channel wireless fNIRS devices. Duan et al. [37] introduced a framework for fNIRS hyperscanning referred to as “cluster imaging of multi-brain networks” (CIMEN) and acquired fNIRS data from a group of nine people during a drum beat synchronization task. Babiloni et al. [38,39] and Astolfi et al. [40] acquired EEG data while four people played a card game, and Babiloni et al. [11,41] also studied professional saxophonists’ group interactions in a musical quartet scenario. Furthermore, multilateral EEG acquisition in the classroom environment has been reported [42,43,44].

Although many-to-many or one-to-many interactions seem more interesting and compelling in hyperscanning studies than do dyadic interactions, there are several reasons why group interaction studies have been reported rarely to date:

- Multiple (at least two or more) acquisition devices are necessary for hyperscanning studies. Depending on the number of channels, reliable commercial EEG or fNIRS devices may range in price from $3000 to over $10,000 per unit. To reduce the cost, it may be necessary to custom-made devices or reduce the number of channels greatly. In addition to their cost, recruiting participants and managing experiments with many participants are not easy. As the number of participants increases, the total experimental cost, including management cost (the number of staff, device maintenance, preparation time for the experiment, and so on) increases significantly.
- Because of these problems, no well-controlled experimental paradigm for many participants (more than two) has been developed. To mimic daily life interactions in hyperscanning experiments, participants need to interact freely and easily without feeling they are controlled. However, the data acquired are likely to include severe artifacts, such as movement noise, when great freedom is given during the interaction task. It is relatively easy to identify features or analyze data under well-controlled experiments, such as a locked task time and synchronized stimulus presentation. These two factors (realistic experimental settings and ability to conduct data analysis easily) may lead to problems that make it difficult to satisfy both factors. Furthermore, unlike dyadic interactions, there may be exponentially greater degrees of control in group interactions, and thus, designing a well-controlled experimental paradigm is very challenging.
- It also is difficult to develop multilateral analysis techniques. Connectivity and causality are used primarily in analysis in hyperscanning studies. In the case in which three or more people interact, the inter-relation between a pair of people can be far more complex than in a dyadic interaction, and existing connectivity techniques may be unsuitable for multilateral analysis.
- Multilateral commercial acquisition systems for hyperscanning are not popular in the market. Each manufacturer may provide its own solution for hyperscanning, but it is difficult to measure many (more than two) people at the same time, and the data acquisition strategy for hyperscanning differs depending on the manufacturer [45]. To the best of our knowledge, there is no commercial hyperscanning acquisition system for more than four people. Furthermore, it is difficult to find detailed reports on the way to set up and implement such an acquisition system.

Many other issues remain unresolved in multilateral hyperscanning studies, and it is believed that the first priority is to develop a framework that acquires biosignals from many people simultaneously. With respect to simultaneous EEG acquisition from many people, simple channel division from a few high-density EEG systems is not applicable, because EEG requires its own reference for each person. Thus, it is essential to use one EEG device for each person. In that sense, some multilateral EEG systems have been commercialized recently. BioSemi devices can support simultaneous EEG acquisition for up to four people through a daisy chain method. In one group interaction study [41], four identical EEG devices from EB Neuro were used to connect to interfaces via fiber optics and from interfaces to a commercial switch device via a local area network (LAN). Software the manufacturer provided also was used. These commercial solutions may be highly reliable, and provide hardware synchronization from multiple EEG amplifiers. However, they are quite costly, and may have limited scalability for a large number of participants. Thus, this has motivated investigators to develop a customized framework for a simultaneous acquisition system. Poulsen et al. [42] recorded EEG data from a group of nine people simultaneously using tablets connected to the Emotiv device. Dikker et al. [43] and Bevilacqua et al. [44] used portable EEG devices (Emotiv EPOC+) to acquire data from 12 and 13 people on a single personal computer (PC, MacBook Pro), and used custom-made acquisition software with the openFrameworks software package [46].

There is a high demand to build multilateral EEG acquisition systems, and some recent studies have introduced systems [42,43,44] and various methods to obtain EEG data from multiple people simultaneously. However, to the best of our knowledge, there is little information in the literature that describes the procedural details and technical issues of simultaneous acquisition systems, such as internal algorithms, operating principles, or the open source code required to implement the system. The main purpose of most hyperscanning studies is to investigate the INS, so they are less likely to focus on the system used in the experiment. In addition, custom-made acquisition systems tend to be difficult to apply in various hyperscanning experiments because they likely were developed for specific experiments. Poulsen et al. [42] used an ‘n:n’ connection and reported that they generated an electric spark for synchronization. However, it is difficult to monitor data or present events in real time in this structure. Dikker et al. [43] and Bevilacqua et al. [44] addressed the use of the openFramework platform for system development. However, although openFramework is a very large, general-purpose software platform, it is unsuitable for real-time neuroscience. Therefore, it has not been reported whether the acquisition system was able to perform the synchronization, event trigger presentation, real-time monitoring, and processing necessary for the experiment. Furthermore, they did not address specifically the way the system was constructed or the way it communicated, and whether the various functions required for the experiment were possible. This motivated us to introduce our recent custom-made EEG acquisition system.

In this work, we propose our framework for simultaneous EEG acquisition and discuss in detail our various trial-and-error experiences while developing the system. We used OpenViBE, a platform for real-time neuroscience, as the software basis to enable functions (e.g., stimulus presentation and real-time monitoring). Our proposed framework also can be used for a variety of hyperscanning experiments, not just one specific experiment. In addition, we proposed a strategy to synchronize devices and reduce the hardware load. A detailed comparison and summary of existing systems is given in Table 1. As mentioned above, little detailed information about the framework has been addressed in the existing research, but this work describes the framework’s structure and internal operation principle to increase replicability. Section 2 discusses three methods to connect devices in a multilateral simultaneous system and introduces both our custom-made system’s hardware and software aspects. In Section 3, we investigated the way our proposed acquisition system worked by estimating the acquisition delay and signal quality with event-related potential (ERP) experimental data acquired from a group of nine people. Finally, we report all of the technical issues associated with the simultaneous acquisition system in Section 4.

## 2. Acquisition Framework Development

### 2.1. Connection of Devices 

**Figure 1:**
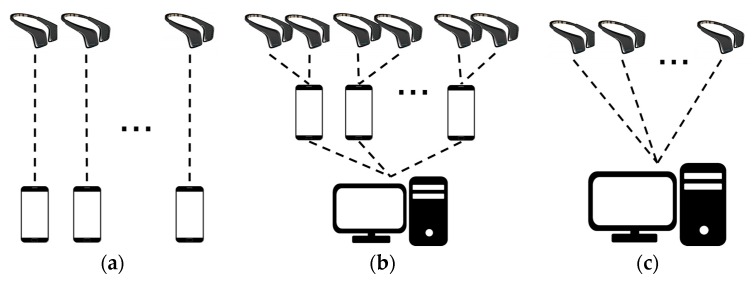
Three ways to connect multiple devices.

As mentioned earlier, the modality used for large-scale hyperscanning is likely to be fNIRS or EEG, both of which are portable and cost-effective. In this work, we proposed a large-scale hyperscanning framework using EEG devices. There are several ways to connect multiple devices to the server, and real-time data acquisition architectures largely are categorized in three ways, as Figure 1 illustrates:

First, each device is connected individually to each low-specification computer (Raspberry Pi, Arduino, tablet, PC, etc.), as shown in Figure 1a and is referred to as the ‘n:n’ connection for n devices.

Second, data are sent in two steps. In the first, each group of k devices among n (= k×p) devices is connected to one low-specification server; then, in the second, p low-specification servers are all connected to one high-specification server, which is referred to as the ‘k:p:1’ connection for n devices; Figure 1b shows the case of k = 2.

Third, all devices are connected directly to one high-end server simultaneously, as depicted in Figure 1c, which is referred to as the ‘n:1’ connection for n devices.

Among these three, we adopted the last—the ‘n:1’ connection (Figure 1c)—as our proposed acquisition framework. We observed that the ‘n:1’ connection is advantageous in the experimental procedure because it has a short communication delay, and allows direct control of the device, as well as real-time signal acquisition, monitoring, and processing.

**Figure 2:**
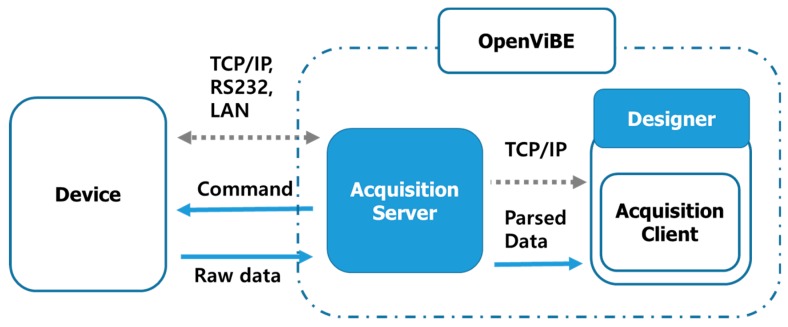
OpenViBE working flow. Dotted arrows (in gray) indicate communication protocol, solid arrows (blue) indicate data flow, and colored boxes represent processes.

Hyperscanning experiments require not only simultaneous data acquisition from multiple devices, but also various functions for experimental progress. Fortunately, there are well-organized open sources for real-time experiments, such as BCI2000 [49], OpenViBE [47], and BCILAB [50]. We adopted OpenViBE in our proposed acquisition framework because it has synchronization and multi-acquisition modules for multimodality. OpenViBE is open-source software for real-time neuroscientific experiments developed in INRIA. It provides many functions, such as scripting, real-time signal monitoring, acquisition, filtering, stimulus presentation, and file input/output. The basic structure and acquisition flow between OpenViBE and the device are illustrated in Figure 2. The acquisition server controls the device, receives data from it, and parses them. The designer communicates with the acquisition server and performs functions to process the data, such as real-time monitoring, filtering, and file input/output. Our custom-made code used to control the device, synchronize all devices, and finally acquire EEG data, was added to the OpenViBE acquisition server.

### 2.2. Hardware

**Figure 3:**
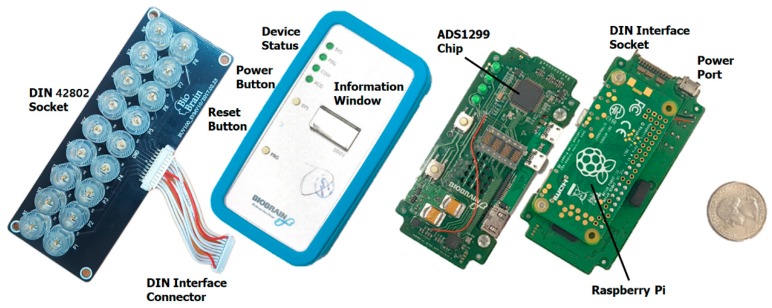
Our custom-made device for the multilateral EEG acquisition system.

Firstly, a small piece of EEG acquisition hardware was developed and manufactured for our multi-user (large scale) hyperscanning (BioBrain Inc., Daejeon 35203, Korea). An ADS1299 chip from Texas Instruments (Dallas, TX, USA) was used for the EEG acquisition module, and 2.4 GHz Wi-Fi was introduced to communicate via the TCP/IP software protocol. The Raspberry Pi Zero W model was adopted as the wireless communication hardware. This EEG device, which weighs approximately 56 g (without battery) and is 93 × 47 × 16 mm in size, is battery-powered, operates at a speed of 1 kHz, and consists of seven monopolar and one bipolar channels. We note that a 10,000 mA Xiaomi battery was used (approximately 194 g) in our validation experiment, as our device must operate continuously for up to two hours. EEG data are transmitted via a commercialized wireless router (AD7200-Nighthawk^®^ X10 model from NETGEAR). Each device is connected to the router and assigned to one internet protocol (IP) address. In addition, our manufactured device uses a Deutsches Institut für Normung (DIN, German Institute for Standardization) 42802 type snap-lead and a passive disposable foam electrode. The header consists of a synchronization element (2 bytes), device information (1 byte), and 3 bytes of the packet number as a timestamp. The payloads are a total of 24 bytes with 8 channels of 3 bytes (24-bit resolution per channel). Therefore, one device’s transmission rate is 30 kB per second. Our hardware for wiring and the electrodes is shown in Figure 3. 

### 2.3. Software

**Figure 4:**
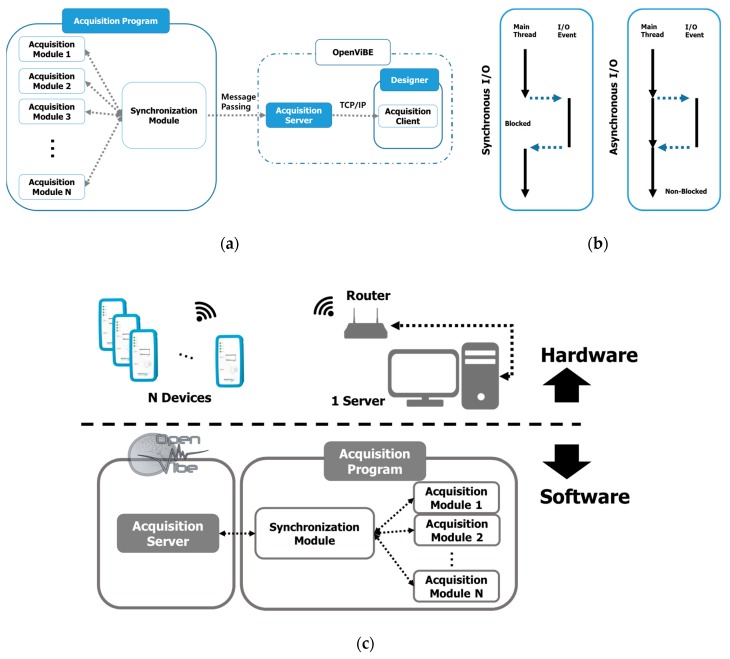
(**a**) Connection diagram of acquisition program and OpenViBE. Our custom-made software acquires data from the devices and sends parsed and synchronized data to the OpenViBE acquisition server by message passing; (**b**) Comparison between synchronous and asynchronous communications. In synchronous communication, the main thread should wait until data from the device are read; however, asynchronous communication delivers the transmission request only and continues to process the main thread; (**c**) Diagram of framework configuration overall.

Acquisition devices and processes were connected as a pair in the OpenViBE data acquisition module. This structure may exert a heavy load on the system because the number of processes increases with the number of devices. Thus, we introduced asynchronous communication, as shown in Figure 4b. In our proposed architecture, communication consists of two phases—device/ software (custom-made) and software (custom-made)/acquisition server. Our custom-made software performs parsing, merging, and synchronizing tasks, and sends the combined EEG data to the acquisition server by the message passing method. The acquisition server parses the data received into the correct block size for OpenViBE and sends it to the designer (Figure 4a). We note that at the current level, data can be acquired from up to 20 devices simultaneously.

## 3. Validation of our Designed Framework

### 3.1. Validation Experiments 

#### 3.1.1. Communication

We attempted to measure the communication delay in the structure of our proposed framework for a multilateral EEG acquisition system. Communication delay was estimated with the ping utility, which measures the round-trip travel time of the message sent from the host to the target. We defined the wireless transmission delay as half of the ping result (one-way travel time). The parameters that influence the transmission speed are presumed to be the number of devices connected and their mean distance from the router. Thus, we measured the communication delays as the two parameters varied. Keeping the devices turned on and connected to the server PC, a pulse signal was sent to each device every 10 ms after setting the sending buffer size to 32 bytes and the timeout to 500 ms. For each test, a total of 1000 pulses was sent to estimate delay. This test was repeated 10 times, and finally, the communication delay was estimated by averaging the delay time over 10,000 pulses and computing the loss rate. The two cases we tested are as follows:

- Case 1: The number of devices connected varied from 1–10 and each device was kept 3 m from the router
- Case 2: With 10 devices connected to the router, the communication delay was estimated over various distances (2.0, 2.5, 3.0, 3.5, 4.0, 4.5, 5.0 m) between the devices and router

For each case, the final result was the grand mean of the communication delays over all devices. This test was performed on a PC (Intel^®^ Core ™ i7-6700k central processing unit (CPU), GeForce 1080 graphics adapter, 32 GB double data rate fourth generation (DDR4) memory, Windows 10 operating system (OS)). We note that no program that used the network was run during the test except the ping tool.

#### 3.1.2. Event-Related Potential Experiment

It is necessary to verify that the framework proposed in this work can acquire multiple EEG data simultaneously. Thus, ERP experiments were conducted for verification purposes because ERP is highly sensitive to the time at which stimuli are presented. Therefore, it is necessary to know whether the devices can be synchronized reasonably well according to the given triggers. Under the conventional ERP experimental paradigm, a group of nine people participated together and EEG data from three channels (Fp1, Fpz, and Fp2) were collected simultaneously from all. Reference and ground channels were placed on both mastoids and all other channels were fixed to the scalp with disposable sticker-type electrodes without a cap. All subjects gave their informed consent before they participated in the study, which the institutional review board of the Gwangju Institute of Science and Technology approved (20160120-HR-21-01-02). The detailed experimental paradigm is as follows.

**Figure 5:**
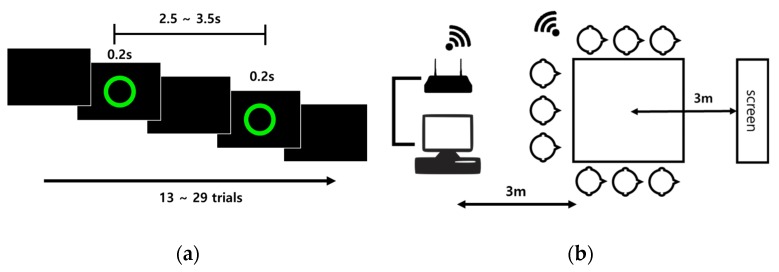
(**a**) Visual ERP paradigm; (**b**) Experimental configurations. Nine people participated simultaneously in this group experiment. They sat approximately 3 m away from the monitor and were instructed to focus on and count the visual stimuli.

The green circular stimulus for the visual ERP is presented on a blank black background on a screen, as shown in Figure 5a. All participants sat 3 m on average from the screen, and were instructed to count the number of visual stimuli presented. All stimulus onset times were marked for the visual ERP. An inter-stimulus interval (ISI) between 2.5 s and 3.5 s was selected randomly and the stimulus duration was 0.2 s. A range of 13–29 trials were conducted for each run, and 10 runs were conducted for a total of 188 trials collected for each participant. 

The ERP component is difficult to detect with only one or several trials. Generally, as many as tens or hundreds trials are averaged to detect the ERP component and estimate the ERP P300’s amplitude. Tens of trials (at least) are necessary to acquire EEG from one person and detect an ERP component. We hypothesized that if the simultaneous EEG acquisition system worked reasonably well—i.e., synchronizing nine devices was sufficient—all EEG trials acquired from multiple people under the experimental paradigm may be used to generate the visual ERP component, thereby reducing the number of trials required to detect ERP greatly. To test this hypothesis, we defined ERP detection in this experiment when all three of the following conditions for the ERP component were met:

- Its minimum peak appeared 140–300 ms after stimulus onset.
- Its maximum peak appeared 280–450 ms after stimulus onset.
- Its peak to peak amplitude was greater than 6 μV.

We note that these conditions were determined empirically. Based on the definition of ERP detectability above, we counted the number of trials each individual participant required to detect the ERP component and investigated ERP detection as the number of trials increased from 6 to 60. For example, to determine the detectability during 10 trials, we computed the first mean for the first to 10th trials, the second for the second to 11th trials, and so on. This was computed continuously until the last trial, which was used as the mean. Finally, the detection rate was estimated from all means for the successive trials in the n trials given (n varied from 6 to 60). In this way, we estimated each individual and the group of people’s detection rates, respectively. We note that one trial in the group analysis was multiple trials depending on the number of people (one acquired from each person).

The EEG data acquired were band-passed between 1 and 15 Hz (Butterworth). Each trial was cut temporally [-200, 1000] ms according to the visual stimulus onset. A temporal window of 200 ms before stimulus presentation was used as the baseline correction. Trials that exceeded an amplitude of 60 μV were rejected automatically as bad trials. We also rejected trials associated strongly with eye movement, blinking, and other artifacts by visual inspection. These rejected trials were not used to detect ERP, but their number was counted. We observed that the number of trials removed because of artifacts varied from person to person. We note that it was difficult to apply artifact removal techniques, such as independent component analysis (ICA), for data from a small number of channels.

### 3.2. Validation Results 

Ping yields the approximate round-trip time for the message sent from the host to the target. As addressed in the previous section, communication delay was defined as half the ping time. Table 2 shows the estimated communication delays when the number of devices varied while the distance from the router was fixed 3 m away. We observed that the ping time nearly always was approximately 3 ms regardless of the number of devices connected. Furthermore, regardless of the number of devices connected, the loss rate was between 0.65–1.34%, which is negligible. Table 3 presents the communication delays over various distances between the device and router when 10 devices were connected. The distance varied from 2 to 5 m in 0.5 m increments. We found that there was no notable difference in delay within a distance of 5 m. It is quite interesting that 10 devices (participants) all may be located easily in a room within 5 m away from the router during the experiment. Thus, in practice, all EEG data acquired reached the server within 1.5–2.0 ms.

**Figure 6:**
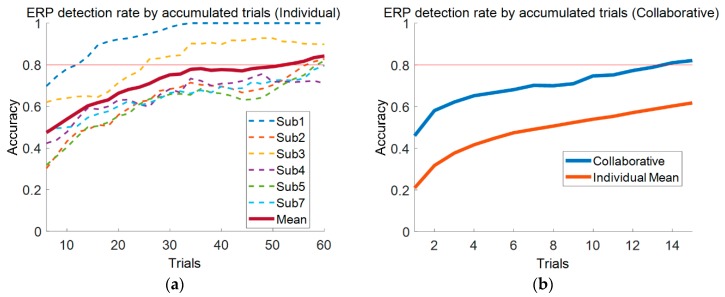
ERP detection rates as the number of trials increased. (**a**) Individuals’ ERP detection rates. The red bold curve represents the mean detection rate over all participants; (**b**) The ERP detection rate for the group of six participants (all of three participants’ ERP trials were rejected, so six participants’ trials were used ultimately).

We collected 188 trials for each participant in the ERP experiments, and observed that the number of bad trials varied considerably among them. We found that two participants (Sub2 and Sub5) showed a relatively smaller number of good trials than did the others because eye blinking and stimulus presentation overlapped very frequently. Three participants’ (Sub6, Sub8, and Sub9) ERP data were eliminated from our analysis because of unusual patterns. The details are discussed in Section 4.3. Ultimately, six participants’ ERP data were used in the individual and group analyses. Figure 6 represents the ERP component’s detection rates at Fpz as the number of trials increased. Individual participant’s ERP detection rates and that of the group were compared. In the group case specifically, we gathered six synchronized trials from six participants simultaneously to generate the ERP component. For example, 4 trials in the group ERP test represented 24 trials (4 from each participant). All cases showed clear moderate increases in ERP detection rates as the number of trials increased. In individual cases, most participants required over 54 trials to yield an ERP detection rate of 80% or above, while 14 trials were sufficiently good to yield an ERP detection rate of 80% for the group case. We note that all EEG trials of three of the nine participants were eliminated from the group analysis; thus, the group ERP detection required approximately 1/5 the number of trials for individual ERP detection.

## 4. Discussion

### 4.1. Connection and Synchronization of Devices

The simplest way to acquire data simultaneously is to use as many computers as devices, as shown in Figure 1a. This ‘n:n’ connection, without specific implementation, may be available with the commercial software the manufacturer provides, and is easy to implement even when software development is required. However, we knew that it is difficult to synchronize the devices, send triggers, and present stimuli during an experiment with the ‘n:n’ connection. Poulsen et al. [42] introduced this connection in an experiment with a group of nine people, and noted that the electric spark was used for synchronization. It is said that a piezoelectric spark generator produces a strong spark up to 60 kV, but it was not visible in EEG. Synchronization may be possible if a spark generator is used to make a strong spark, as it may allow synchronization at the beginning or end of the experiment. However, synchronization during the experiment relies completely on the devices and it is not easy to mark the trigger or timestamp during the experiment. Thus, the other two methods, the ‘k:p:1’ (n = k × p) or ‘n:1’ connections (Figure 1b,c) are more preferable when the experiment requires sensitive timestamps, stimulus presentation, and feedback. Comparing the ‘k:p:1’ and ‘n:1’ connections, ‘k:p:1’ may be less advantageous with respect to communication delay, because data are transmitted in two stages, and their synchronization may be a hurdle. As the number of ‘p’ servers increases, and the load on the central server definitely is reduced significantly because of the intermediate servers’ processing. While the ‘n:1’ connection may control the device on the server directly and it is simple to send commands and minimize communication delay, it may exert a heavy workload on the central server.

It is known that the ideal approach to synchronize the EEG acquisition system is to use synchronization hardware in a wired environment. Hardware-based synchronization is advantageous, in that it may minimize unavoidable communication delays in a high-speed environment. Generally, however, it is too expense to manufacture and the cost increases dramatically with the number of participants. Although a wired connection between the device and server is used widely in practice, it is quite inconvenient when a hyperscanning experiment is conducted that acquires data from many people simultaneously. Thus, to allow the participants more mobility, a wireless connection is used most often today. Duan et al. [37] reported that participants’ movement was difficult to accommodate because of the limited length of optical cables in fNIRS device, and similar problems may be encountered in any kind of wired environment. This limits the participants’ position and motion, and the experimental paradigm’s design. As the number of participants increases, the number of cables laying on the floor also increases, which is highly inconvenient when moving to offer experimental guidance and check electrodes. Furthermore, wired devices make it inconvenient to set up the experimental environment. Thus, the cost of conducting the experiment increases with the number of participants. Accordingly, it is highly important for the experimental setup process to be as simple as possible. In this work, to develop a wearable (wireless) system at a reasonable cost, we considered software synchronization for multiple wireless connections. We believe that our proposed system is more convenient to use (because of wireless connection) as well as affordable.

### 4.2. Other Platforms for Multilateral Systems: Lab Streaming Layer

Recently, investigators have considered the software platform Lab Streaming Layer (LSL) [51] for simultaneous acquisition. LSL is software for the unified collection of measurement time series in research experiments that Swartz Center for Computational Neuroscience, USA developed. It is open-source software and supports many powerful functions for real-time research experiments, such as sending trigger events, presenting stimuli, connecting to signal acquisition devices, and capturing keyboard/mouse events. Most importantly, LSL supports time synchronization designed after the Network Time Protocol (NTP) [52], and may be applied potentially to large-scale connections. However, we found that LSL could not maintain the structure we proposed, as it largely is structured for a dyadic connection (between sender and receiver); thus, it is simple to implement to execute one process for each device, which is identical to the ‘n:n’ connection (Figure 1a). As addressed in Section 2, this connection may require much greater consumption of hardware resources in OpenViBE-based multi-process architecture when 10 or more acquisition processes are executed simultaneously. To solve this problem, we proposed a framework that introduced a multi-client centralized connection approach that reduces the number of processes to one.

Although it appeared that LSL cannot be applied to our structure easily, LSL and OpenViBE acquisition servers may differ in resource consumption during processes. The hardware load may not be significant in LSL while processes are maintained. Furthermore, it may attempt to reduce loads by using multi-thread. Thus, LSL is a good alternative for large-scale systems that support strong time synchronization and various functions, although to the best of our knowledge, there is no report on large-scale EEG acquisition via LSL. Thus, to verify its usefulness in multilateral acquisition systems, extensive and intensive investigation may be needed, which will be performed in our subsequent work.

### 4.3. Recent Version of OpenViBE

Our proposed framework’s basis is OpenViBE v. 1.3.1. However, this is updated regularly and the most recent version is 2.2.0. We found that there were three major updates, but the news release did not report any improvement in the acquisition server’s resource consumption. Because OpenViBE has reported continuous performance improvements in every update thus far, there may be some progress in acquisition load and a newer version may be able to acquire EEG data more stably. Thus, we note that the latest version OpenViBE needs to be tested and implemented.

### 4.4. System Load

Most work to date has been conducted with the ‘n:n’ connection structure, which is the one-to-one connection between OpenViBE and a single device. In this case, to achieve simultaneous acquisition, the server may need as many real-time processes as the number of devices. One of the ‘n:n’ connection structure’s hurdles is its high hardware load. As the number of acquisition server processes increases, CPU use may reach nearly 100%, which prevents the server from functioning. The acquisition server, designer, and synchronization processes all are conducted in real-time and require continuous processing of the data the devices send; the load is high because the process usually continues without idle time. In the case in which only the acquisition server is run, we observed that there are no resources available when a dozen or more processes are activated. When the designer and acquisition processes were executed together and data were acquired, the CPU use rate reached 100% and function failure. Because a designer process with multiple functions, such as monitoring and filtering, is needed during these experiments, we observed that more than eight connections are intractable in the ‘n:n’ connection structure. We note that all tests were performed on a PC (Intel^®^ Core ™ i7-6700k CPU, GeForce 1080 graphics adapter, 32GB DDR4 memory, Windows 10 OS, with OpenViBE v. 1.3.1).

Two schemes were introduced in our proposed architecture to reduce the number of processes and reduce the hardware load thereby.

- A server process was connected with multiple clients (devices). As a unit of program execution, the process was allocated a unique address space and hardware resources, including CPU registers, text data in memory, and open files and devices. Therefore, as the process increases, the burden on resources increases. In addition, as the server’s OS does not run just one program, it is necessary to change the process that it executes, which entails a very high cost in context exchange. Therefore, we made it possible for the software to communicate with all devices in one process that reduced the number of processes overall.
- Second, asynchronous communication. There are synchronous and asynchronous modes of communication, as shown in Figure 4. In the synchronous communication mode, CPU blocks and no other operations can be processed until a response arrives from the device. In this structure, a greater number of devices increases the blocked time required for input/output (I/O), which may result in very inefficient operation. However, asynchronous communication reduces the hardware resource load dramatically, even in situations in which multiple devices are connected. Nonetheless, in asynchronous communication, the OS determines the send timing and the data’s size, so it is necessary to parse the data and control synchronization between devices.

Our custom-made device operates on 8 channels at a sampling frequency of 1000 Hz. Based on the device developed, data may be acquired from up to 20 devices using the current framework. However, it is advantageous for EEG devices to have as many channels as possible in hyperscanning studies because various cortical areas’ involvement can be investigated. It is natural to question whether the number of devices in a simultaneous acquisition system should decrease when each device’s number of channels increases. A practical test is required to confirm this, as we expect that a mild increase in the number of channels (addition of just several channels per device) may not lead to any problems and maintain the number of connections unless the number of channels increases dramatically. It is understood that the current load problem on the hardware is not the amount of transmission on the network from the device, but the CPU resource consumption. For example, we tested a 64 channels Biosemi ActiveTwo device using the one-to-one communication method on the same server. Biosemi operates at a sampling frequency of 1024 Hz, and our 8-channel device operates at a sampling frequency of 1000 Hz. A fair comparison is not even possible, as with respect to the amount of transmission from the device to the server, Biosemi is expected to transmit the roughly same amount of data as that of the eight devices we developed. However, in practice, compared to hardware consumption, Biosemi consumed less than 30% of the CPU’s resources, while eight simultaneous connections did not operate normally because 100% of the CPU’s resources were consumed. It was inferred from this observation that network traffic may not cause the hardware load, but context switching for execution between many real-time processes. Such an understanding and observation motivated us to propose our simultaneous acquisition framework with the single process structure and asynchronous communication method.

### 4.5. Electrodes 

**Figure 7:**
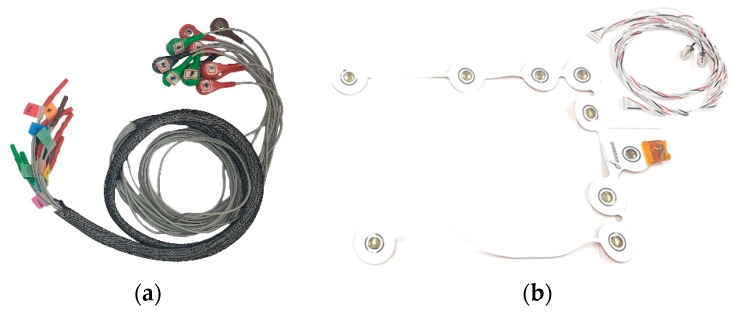
(**a**) Appearance of the DIN 42802 snap-lead; (**b**) Flexible PCB we developed.

A large number of people participated in our experiment; thus, we found that it took quite a long time to prepare the experiment. First, sticker-type disposable electrodes were made that were more convenient for the participants. They were pre-applied with a conductive gel, which reduced the preparation time for the experiment and were easy to use because they are disposable. Furthermore, they can be used without EEG caps or pastes, which also reduced the time to prepare the experiment greatly. However, we found that they could not be attached to areas of the scalp with hair because they need to be attached directly to the skin; thus, they were used on the frontal lobe only. The terminal at the opposite end of the snap-lead (shown in Figure 7a) is a DIN 42802 type used widely and is compatible with all passive types of electrodes: dry, clip, disk-type (used with glue), and so on. Therefore, the electrode’s type and position can be selected freely according to the experiment’s purpose, and the EEG cap also may be used for its convenience. In addition, ear clip electrodes can be used as a reference for signal quality if other electrodes need to be placed near the mastoid.

Our custom-made devices are compact and mobile; however, participants still reported discomfort during the experiment that was found to derive from the DIN 42802 snap-lead. The leads became twisted and because the line weighs over 150 g, including all channels (reference and bipolar), they are heavy to use over a long period. To compensate for this inconvenience, we considered a flexible printed circuit board (PCB), as illustrated in Figure 7b. This PCB is lighter in weight and twists less when used, which makes it considerably more convenient than the previous DIN 42802 snap-lead. In any case, both commercial and research parties continue to make great efforts to improve all aspects of electrodes (convenience, weight, cost, and mobility).

For several reasons, the cost required to prepare and execute multilateral hyperscanning experiments is very high. One of the important reasons is that EEG data should be obtained using a conductive gel or paste to fix the electrode to the scalp. In this work, sticker-type electrodes were used for convenience; however, they had some limitations, as EEG data could be obtained only on the frontal area, and they detached easily and frequently because of subjects’ sweat or facial movement. Dry electrodes, which do not require conductive gels or pastes, and unlike the sticker-type, can be used on all areas of the scalp, may overcome these disadvantages. Fortunately, the electrode industry has developed dry electrodes in recent years that perform well, and some comparison studies have been conducted to verify their usefulness [53,54]. We expect that dry electrodes will be more reliable and comfortable, and have many advantages in time and cost in preparation for experiments, and thus, may be a good choice for multilateral hyperscanning experiments.

### 4.6. ERP Detection

**Figure 8:**
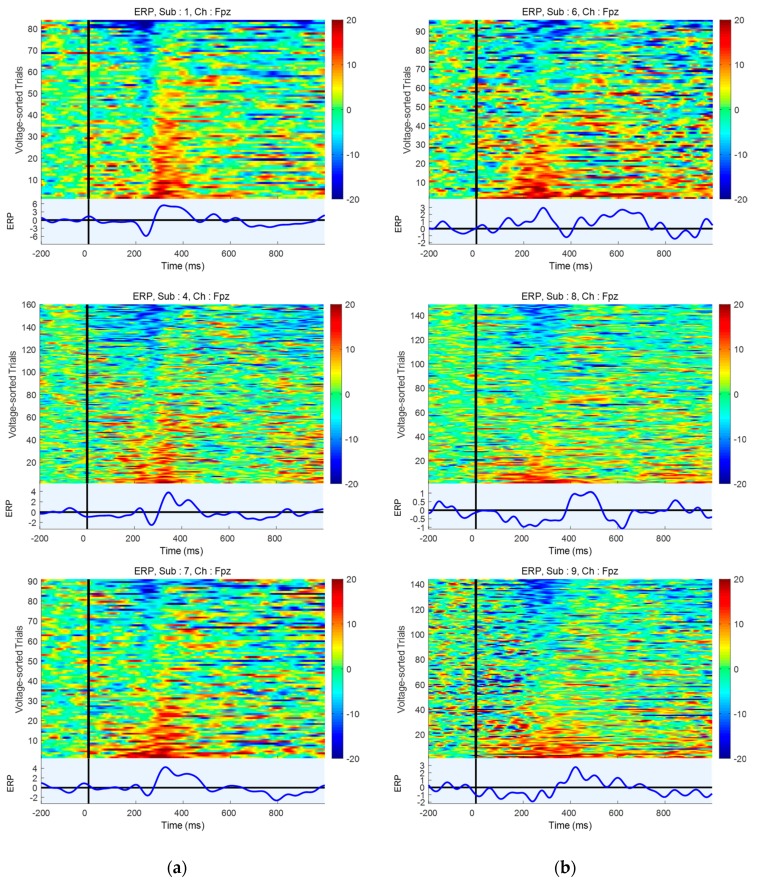
Colormap of ERP data over trials and ERP component by averaging over trials. (**a**) Cases for participants who performed well; (**b**) Cases for participants who performed poorly.

With our proposed system, we acquired ERP data from nine participants for validation purposes, as illustrated in Figure 8. The trials were sorted (in decreasing order) according to the mean amplitude between 200 and 350 ms. Six participants yielded reasonable ERP components. However, N2 and P3 components (typical components of visual ERP) largely were absent in three participants’ (Sub6, Sub8, and Sub9) ERP data, which is illustrated in Figure 8b. Compared to the ERP cases of the participants who performed well (Figure 8a), N2 components were not seen clearly, and even P3 components seemingly appeared 400 or more ms after onset. Thus, we could not confirm that they are typical ERP components. We presumed that subjects’ characteristics were one possible reason for this outcome. However, although we excluded these three subjects’ data from our analysis because of unusual ERP observations, by checking trigger indications, we did confirm that simultaneous acquisition under our framework was successful.

In practice, ERP components cannot be detected clearly from a single trial; thus, as many trials as possible are averaged until the component appears clearly. Acquiring a great number of trials takes time and tires participants. This motivated us to investigate whether acquiring ERP data from a group of people simultaneously can reduce the number of trials required for ERP detection by using all participants’ trials combined. Using trials from a group of people to achieve ERP detection may be quite interesting and ERP may be detectable with far fewer trials. It is believed that this application will reduce the training process in brain–computer interface (BCI), such as P300 spellers, dramatically. In this study, sticker-type disposable electrodes were used and EEG data were acquired from the frontal lobe only. Thus, we observed that the EEG data acquired were quite noisy because of eye blinking and rolling, and the three participants whose data were excluded experienced this to a greater degree.

Excluding these three participants, we tested ERP detection from six participants combined. We found that individual participants required at least 54 trials to achieve better than 80% performance in the ERP detection rate. However, even when only six participants performed the same task simultaneously and their ERP detection trials were used in combination, the number of trials required to detect ERP decreased dramatically (from 54 to 14 to achieve 80% detection performance). These results confirmed that our proposed system works reasonably well in a hyperscanning experiment.

### 4.7. Applications, Limitations, and Future Work

Our proposed system can be applied to group interaction studies in neuroscience, and will allow us to design experimental paradigms for hyperscanning more freely. More realistic experimental settings, such as discussions, presentations, and classroom situations may be tractable to investigation. It also allows us to conduct research on choices and interactions in social contexts, such as the Asch paradigm [55]. We expect that the framework can be used to evaluate the interaction between teachers and learners to improve our understanding of learning’s effectiveness. Furthermore, it is possible to evaluate video content or produce audience-interactive content through biomarkers from a large number of people. It also may be applied to the development of BCI technology. BCI is a technology that uses brain signals to communicate the user’s intentions to a computer without the need for any input devices such as a keyboard and mouse. BCI technology has been applied to spellers for quadriplegic patients, prosthesis and environment control, and rehabilitation aids. Among them, a collaborative BCI, which may be a method to overcome BCI’s limitations, can be combined with hyperscanning technology to improve BCI performance [56,57] or assist patients [58]. Particularly, a collaborative BCI and guided BCI paradigms are under development.

There are some limitations in this work, as follows:

- The framework proposed adopted a connection using a 2.4 GHz Wi-Fi. Generally, a 2.4 GHz band is used in many devices, such as wireless keyboards and routers. Therefore, devices operate stably when there is no other router or device nearby that uses 2.4 GHz. However, communication disconnection or delay may occur when the conditions surrounding the wireless connections are crowded.
- Ping was used to estimate the transmission delay, which is technically different from the EEG data devices send, and therefore it may offer only a rough estimation. In addition, the ping tool’s accuracy is limited inherently because of its temporal resolution in ms.
- A very simple ERP component that has a minimum/maximum peak in the N2 and P3 ranges was investigated in the validation experiment. However, N2 and P3 timings vary greatly depending on the trials or people. For example, one participant (Sub 4) showed clean EEG data in which it was easy to detect the ERP component (Figure 8a). However, the ERP component’s shape did not meet the criteria we proposed and thus, it did not perform well. Therefore, we believe that other criteria for ERP detection may yield different or better results.
- In this work, our framework was tested with a group of nine people. However, we confirmed that it could work with up to 20 people. Because of usable devices’ limitation during experiments, only nine devices were used. We will continue to perform experiments with larger groups in the future.

In this work, we implemented a multilateral acquisition framework without a sophisticated experimental design and INS analysis for group interaction. Group interactions under our framework are currently under investigation.

## 5. Conclusions

There is a demand for, and research efforts on, group interactions. However, the methodology has not been discussed in detail. Thus, in this study, a framework for hyperscanning was designed and developed that allows EEG data to be acquired simultaneously from a group of people (over 10 and up to 20). Furthermore, various issues we encountered and solutions we found while developing our proposed system were discussed. We also conducted an ERP experiment to verify our system, and found that time-sensitive ERP components were obtained successfully with EEG data recorded simultaneously from a group of nine people. We confirmed that the multilateral acquisition system has the potential to reduce the trials required to detect ERP components and suggests the possibility of various applications, such as collaborative, assisted, and multi-mind BCI, brain gaming, crowd psychology, and interactive contents development.

## Figures and Tables

**Table 1:** Comparison to other custom-developed EEG acquisition frameworks.

Device	Wireless EEG [42] (Emotiv EPOC)	Wireless EEG [43,44] (Emotiv EPOC+)	Wireless EEG (Our Custom-Made System)
**Sampling Frequency (Hz)**	128	256	1000
**Number of Channels**	14	14	8
**Concurrent Acquisition (Maximum)**	9 (N/M ^1^)	13 (N/M)	9 (20)
**Connection Method**	n:n	1:n	1:n
**Basis Software**	-	openFrameworks [46]	OpenViBE [47]
**Synchronization during Experiment**	Electrical spark	N/M	Frame number, synchronization module
**Framework Structure**	-	N/M	Described
**Real-Time Processing ^2^**	N/M	N/M	Possible
**Validation**	N/M	Total independence [48], auditory ERP	Delay, loss rate, visual ERP

^1^ Not mentioned; ^2^ Real-time function including signal monitoring, sending event trigger, present stimulus, etc.

**Table 2:** Estimated communication delay over various numbers of connected devices. The distance from the router to all devices was maintained at 3 m.

**Number of Connected Devices**	1	2	3	4	5	6	7	8	9	10
**Ping Delay (ms)**	3	3	3	3	3	3	3	4	3	3
**Loss Rate (%)**	0.87	0.74	0.79	0.65	0.7	1.34	1.32	1.34	0.86	0.94

**Table 3:** Estimated communication delay over various distances from the router when 10 devices were connected.

**Distance from the Router (m)**	2	2.5	3	3.5	4	4.5	5
**Ping Delay (ms)**	3	2	3	3	3	3	3
**Loss Rate (%)**	0.80	0.62	0.70	0.89	0.76	0.58	0.61

## Abbreviations

INS	Interpersonal neural synchronization
fMRI	Functional magnetic resonance imaging
fNIRS	Functional near-infrared spectroscopy
MEG	Magnetoencephalography
EEG	Electroencephalography
LAN	Local area network
PC	Personal computer
ERP	Event-related potential
IP	Internet Protocol
DIN	Deutsches Institut für Normung (German Institute for Standardization)
CPU	Central processing unit
DDR4	Double data rate fourth generation
OS	Operating system
ISI	Inter-stimulus interval
ICA	Independent component analysis
LSL	Lab streaming layer
NTP	Network Time Protocol
I/O	Input/output
PCB	Printed circuit board
BCI	Brain-computer interface
